# Association between dietary carbohydrate to fiber ratio and metabolic dysfunction associated fatty liver disease in adults: evidence from the NHANES 2017–2020

**DOI:** 10.1186/s41043-024-00543-1

**Published:** 2024-03-28

**Authors:** Zhenmin Liu, Taiyong Fang

**Affiliations:** Department of Gastroenterology, The Second Affiliated Hospital of Fujian Medical University, Quanzhou, Fujian China

**Keywords:** Diet, Carbohydrate to fiber ratio, Metabolic dysfunction-associated fatty liver disease, Adults, NHANES

## Abstract

This study aimed to explore the association of carbohydrate to fiber ratio (CFR) with metabolic dysfunction-associated fatty liver disease (MAFLD) in adults. In this study, data from the 2 cycles (2017–2018 and 2019–2020) of the NHANES were used. Univariate and multivariate weighted logistic regression analyses were applied to evaluate the association between CFR and MAFLD. Odds ratios (ORs) and 95% confidence levels (CIs) were estimated. Subgroup analysis was further performed in terms of gender, age and comorbidity (diabetes, hypertension). A total of 3180 individuals were included, with 1408 (44.28%) in the non-MAFLD group and 1772 (55.72%) in the MAFLD group. After adjusting different variables, a dietary fiber intake of 11.15–18.40 g was associated with significantly lower odds of MAFLD compared with a fiber intake < 11.15 g (OR = 0.71, 95% CI 0.54–0.93). In contrast to a dietary CFR < 12.58, a CFR > 19.91 was associated with significantly higher odds of MAFLD (OR = 1.57, 95% CI 1.09–2.27). Compared with females with a dietary CFR < 12.58, those with a CFR > 19.91 had significantly increased odds of MAFLD (OR = 1.87, 95% CI 1.29–2.73). Among individuals aged < 65 years, a dietary CFR > 19.91 was associated with higher odds of MAFLD than a dietary CFR < 12.58 (OR = 1.52, 95% CI 1.02–2.25). For participants without diabetes (OR = 1.79, 95% CI 1.26–2.54) or hypertension (OR = 1.93, 95% CI 1.02–3.65), a dietary CFR > 19.91 was associated with elevated odds of MAFLD than a CFR < 12.58. In summary, a higher CFR was associated with significantly greater odds of MAFLD, indicating the negative association between carbohydrate quality and MAFLD. The research would be conducive to metabolic dysfunction-associated fatty liver disease treatment.

## Background

Metabolic dysfunction-associated fatty liver disease (MAFLD), previously known as non-alcoholic fatty liver disease (NAFLD), is a metabolic stress-induced liver injury closely related to insulin resistance and genetic susceptibility, characterized by excessive accumulation of fat in hepatocytes [1, 2]. It is the most common chronic liver disease and influences up to approximately 30% of the global population [3, 4], and the prevalence and severity of MAFLD also increase with age [5, 6]. MAFLD is significantly associated with an increased risk of death [7, 8]. With the prevalence of obesity and metabolic syndrome, as well as the intensification of population aging, the disease burden of MAFLD is increasing [9, 10]. Therefore, actively preventing and treating MAFLD is of great significance for reducing the disease burden.

Diet is an important modifiable influencing factor in the occurrence and development of MAFLD [11]. Carbohydrates are the main source of energy in the human body, and their impact on health has always been of great concern. There is evidence that the quality of carbohydrate intake has a greater impact on chronic diseases than the quantity consumed [12]. Refined grains, potatoes, and sugary beverages are associated with an increased risk of chronic diseases, while minimally processed grains, beans, and fruits are associated with a reduced risk; this can be partially attributed to the differences in the structure of carbohydrates from different food sources that affect postprandial blood glucose and insulin; these foods typically correspond to high levels of dietary fiber, which can have beneficial effects by delaying carbohydrate absorption and acting on the gut microbiota [13, 14]. Based on the balance between carbohydrates and dietary fiber, nutritionists have proposed a simple and practical indicator—carbohydrate to fiber ratio (CFR) to identify carbohydrate quality [14, 15]. Existing studies showed a significant correlation between CFR and the risk of metabolic syndrome and metabolic risk factors [16–18]. A negative correlation was found between dietary fiber intake and NAFLD risk [19]. The association between CFR and MAFLD has not been reported yet. Therefore, it is necessary to explore the association between CFR and MAFLD, in order to provide a certain basis for dietary prevention and control strategies for MAFLD.

This study aimed to investigate the association between CFR and MAFLD using the data from the National Health and Nutrition Examination Survey (NHANES) database.

## Methods

### Study population

This cross-sectional study used data from the 2 cycles (2017–2018 and 2019–2020) of the NHANES. The NHANES, a series of studies designed to assess the health and nutritional status of the nationally representative, non-institutionalized population in the United States, combines interviews and physical examinations, and is approved by the National Center for Health Statistics (NCHS) Research [20]. As the data of the NHANES are de-identified and freely available, this study was exempt from further approval of the local institutional review board. Inclusion criteria: individuals (1) aged ≥ 18 years, (2) with liver ultrasound transient elastography examination, and (3) assessed for MAFLD. Exclusion criteria: individuals (1) with abnormally low or high total energy intake (< 500 kcal/day or > 5000 kcal/day for females, < 500 kcal/day or > 8000 kcal/day for males), or (2) without information on dietary carbohydrates and fiber.

### Assessment of MAFLD

MAFLD was defined as hepatic steatosis confirmed by imaging {controlled attenuation parameter (CAP) > 248 dB/m [21, 22]}, combined with one of the following three conditions: (1) overweight or obesity (BMI ≥ 25 kg/m^2^); (2) type 2 diabetes mellitus (T2DM); (3) metabolic dysfunction. Metabolic dysfunction referred to the presence of two or more risk factors for metabolic abnormalities in underweight or normal weight individuals (BMI < 25 kg/m^2^): (1) waist circumference (WC) ≥ 102 and 88 cm for males and females, respectively; (2) blood pressure ≥ 130/85 mmHg or receiving antihypertensive medication treatment; (3) plasma triglycerides (TG) ≥ 150 mg/dL (1.70 mmol/L) or receiving specific drug treatment; (4) plasma high-density lipoprotein cholesterol (HDL-C) < 40 mg/dL (1.0 mmol/L) for males and < 50 mg/dL (1.3 mmol/L) for females or receiving specific drug treatment; (5) the fasting plasma glucose (FPG) level of 100–125 mg/dL (5.6–6.9 mmol/L) or the postprandial 2-h plasma glucose (2hPG) level of 140–199 mg/dL (7.8–11 mmol/L) or hemoglobin A1c (HbAlc) of 5.7%-6.4% (39–47 mmol/mol) in the pre-diabetes period; (6) Homeostasis Model Assessment of Insulin Resistance (HOMA-IR) ≥ 2.5 [HOMA-IR = fasting blood glucose (mmol/L) × fasting insulin (μU/mL)/22.5]; (7) the plasma level of high-sensitivity C-reactive protein (hsCRP) > 2 mg/L.

### Dietary carbohydrates, fiber and CFR

Dietary intakes of carbohydrates and fiber were evaluated via the first 24-h dietary recall interview and included their supplements. The CFR was the ratio of dietary carbohydrates to fiber. Based on stratification level standards reported in previous related studies [23–26], the dietary carbohydrates, fiber and CFR obtained were divided based on their tertiles.

### Other variables

Data on the following variables were also collected: age (years), gender, race/Hispanic origin (White, Black, and other), education [less than 9th grade/9-11th grade (including 12th grade with no diploma), high school graduate/general education development (GED) or equivalent, some college or associate (AA) degree/college graduate or above], marital status (married/living with partner, widowed/divorced/separated, never married), poverty income ratio (0–1, ≥ 1, unknown), smoking, drinking, physical activity (< 450 and ≥ 450 MET·min/week, and unknown), sedentary time (< 4 and ≥ 4 h/d), diabetes, hypertension, dyslipidemia, alanine aminotransferase (ALT, U/L), alkaline phosphatase (ALP, IU/L), aspartate aminotransferase (AST, U/L), gamma-glutamyl transferase (GGT, IU/L), chronic hepatitis B (negative, positive, and unknown), chronic hepatitis C (negative, positive, and unknown), body mass index (BMI) (overweight/normal weight, overweight/obesity), carbohydrate (< 188.30, 188.30–281.09, > 281.09 g), fiber (< 11.15, 11.15–18.4, > 18.4 g), CFR (< 12.58, 12.58–19.91, > 19.91 g), protein, total fat, and energy (kcal). Smoking was defined as smoking at least 100 cigarettes in life. Drinking was defined as drinking ≥ 15 g/d for women and ≥ 30 g/d for men. Physical activity was converted into energy consumption, where energy consumption (MET·min) = recommended metabolic equivalent (MET) × exercise time of the corresponding activity (min), which was converted into weekly energy consumption. Hypertension was defined as self-reported hypertension or systolic blood pressure ≥ 130 mmHg or diastolic blood pressure ≥ 80 mmHg or taking antihypertensive drugs. Diabetes was defined as fasting blood glucose ≥ 7.0 mmol/L or HbAlc ≥ 6.5% or self-reported diabetes or receiving hypoglycemic treatment. Underweight/normal weight was defined as a BMI < 25 kg/m^2^, and overweight/obesity as a BMI ≥ 25 kg/m^2^.

### Statistical analysis

Measurement data were described as Mean [standard error (SE)], and the independent samples t-test was used for comparison between two groups. Enumeration data were reported as the number of cases and constituent ratio [n (%)], and the Chi-square test was used for inter-group comparison. Multiple imputation was performed for missing data, and sensitivity analysis was conducted to compare data before and after the imputation.

Univariate weighted logistic regression analysis was utilized to investigate the variables associated with MAFLD, and statistically significant variables were selected as covariates. Univariate and multivariate weighted logistic regression analyses were applied to explore the association of dietary carbohydrates, fiber and CFR with the odds of MAFLD. Model I was a univariate model, and Model II was adjusted for age, gender, race, education, marital status, smoking, physical activity, sedentary time, diabetes, hypertension, dyslipidemia, ALT, ALP, AST, GGT, BMI, and energy. Odds ratios (ORs) and 95% confidence levels (CIs) were estimated. Further, subgroup analysis was carried out in terms of gender, age and comorbidity (diabetes, hypertension).

All statistical analyses were conducted using two-sided tests. SAS 9.4 (SAS Institute Inc., Cary, NC, USA) was used for data cleaning, missing value processing, and model statistical analysis. *P* < 0.05 indicated significant differences.

## Results

### Participant characteristics

**Fig. 1:**
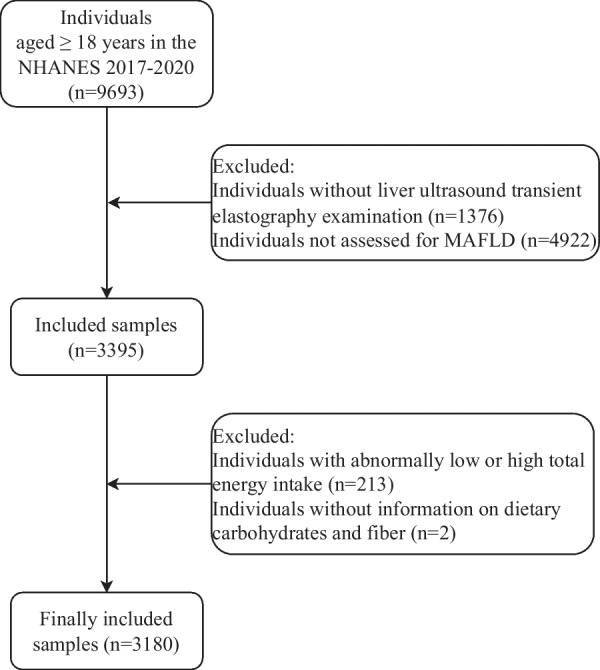
Flow chart of participant selection

A total of 9693 individuals aged ≥ 18 years were enrolled from the NHANES 2017–2020. After excluding individuals without liver ultrasound transient elastography examination (n = 1376), not assessed for MAFLD (n = 4922), with abnormally low or high total energy intake (n = 213), and without information on dietary carbohydrates and fiber (n = 2), 3180 individuals were included in the end, with 1408 (44.28%) in the non-MAFLD group, and 1772 (55.72%) in the MAFLD group. The flow chart of participant selection is shown in Fig. 1. Subjects in the MAFLD group tended to be older (51.25 vs 41.79 years) and males (54.66 vs 46.02%), compared with those in the non-MAFLD group (both *P* < 0.05). There were also significant differences in race, race/Hispanic origin, education, marital status, smoking, physical activity, sedentary time, diabetes, hypertension, dyslipidemia, ALT, ALP, AST, GGT, BMI, and energy between the non-MAFLD and MAFLD groups (all *P* < 0.05). More individuals with MAFLD had diabetes (24.18% vs 4.90%), hypertension (67.34% vs 34.73%) and dyslipidemia (80.39% vs 50.41%) than those without MAFLD (all *P* < 0.05). Table 1 illustrates the baseline characteristics of the study population.

**Table 1:** Baseline characteristics of the study population

Variables	Total (n = 3180)	Non-MAFLD (n = 1408)	MAFLD (n = 1772)	Statistics	*P*
Age (years), Mean (SE)	46.98 (0.74)	41.79 (0.99)	51.25 (0.82)	t = − 8.50	< 0.001
Gender, n (%)				χ^2^ = 9.558	0.002
Male	1605 (50.77)	663 (46.02)	942 (54.66)		
Female	1575 (49.23)	745 (53.98)	830 (45.34)		
Race/Hispanic origin, n (%)				χ^2^ = 11.695	0.003
White	1125 (63.95)	462 (62.98)	663 (64.76)		
Black	812 (10.87)	424 (13.01)	388 (9.11)		
Other	1243 (25.18)	522 (24.01)	721 (26.13)		
Education, n (%)				χ^2^ = 15.844	< 0.001
Less than 9th grade/9–11th grade (including 12th grade with no diploma)	549 (9.92)	217 (8.52)	332 (11.06)		
High school graduate/GED or equivalent	754 (26.26)	320 (23.48)	434 (28.55)		
Some college or AA degree/college graduate or above	1877 (63.82)	871 (68.00)	1006 (60.39)		
Marital status, n (%)				χ^2^ = 32.350	< 0.001
Married/living with partner	1872 (61.54)	760 (53.85)	1112 (67.85)		
Widowed/divorced/separated	692 (19.02)	288 (19.07)	404 (18.98)		
Never married	616 (19.44)	360 (27.08)	256 (13.16)		
Poverty income ratio, n (%)				χ^2^ = 1.904	0.386
0–1	505 (10.52)	229 (9.40)	276 (11.44)		
≥ 1	2271 (79.05)	997 (80.58)	1274 (77.78)		
Unknown	404 (10.43)	182 (10.01)	222 (10.78)		
Smoking, n (%)				χ^2^ = 11.012	0.004
Never	1848 (57.47)	870 (62.15)	978 (53.63)		
Smoking former	751 (26.72)	253 (21.58)	498 (30.94)		
Smoking now	581 (15.81)	285(16.27)	296 (15.43)		
Drinking, n (%)				χ^2^ = 0.422	0.516
No	2762 (82.09)	1228 (82.80)	1534 (81.50)		
Yes	418 (17.91)	180 (17.20)	238 (18.50)		
Physical activity, n (%)				χ^2^ = 9.867	0.007
< 450 MET·min/week	283 (8.12)	123 (6.22)	160 (9.68)		
≥ 450 MET·min/week	2180 (73.82)	1003 (76.65)	1177 (71.50)		
Unknown	717 (18.06)	282 (17.13)	435 (18.82)		
Sedentary time, n (%)				χ^2^ = 4.403	0.036
< 4 h/day	1012 (28.70)	487 (31.76)	525 (26.20)		
≥ 4 h/day	2168 (71.30)	921 (68.24)	1247 (73.80)		
Diabetes, n (%)				χ^2^ = 100.858	< 0.001
No	2508 (84.51)	1292 (95.10)	1216 (75.82)		
Yes	672 (15.49)	116 (4.90)	556 (24.18)		
Hypertension, n (%)				χ^2^ = 115.175	< 0.001
No	1330 (47.36)	802 (65.27)	528 (32.66)		
Yes	1850 (52.64)	606 (34.73)	1244 (67.34)		
Dyslipidemia, n (%)				χ^2^ = 108.862	< 0.001
No	1037 (33.12)	680 (49.59)	357 (19.61)		
Yes	2143 (66.88)	728 (50.41)	1415 (80.39)		
ALT (U/L), mean (SE)	22.66 (0.47)	18.91 (0.59)	25.74 (0.59)	t = − 9.40	< 0.001
ALP (IU/L), mean (SE)	74.90 (0.61)	71.52 (0.86)	77.67 (0.73)	t = − 6.56	< 0.001
AST (U/L), mean (SE)	21.78 (0.38)	20.85 (0.41)	22.54 (0.48)	t = − 3.44	0.002
GGT (IU/L), mean (SE)	28.48 (0.66)	21.62 (0.54)	34.11 (1.05)	t = − 10.70	< 0.001
Chronic hepatitis B, n (%)				χ^2^ = 0.720	0.698
Negative	234 (4.49)	106 (4.34)	128 (4.62)		
Positive	19 (0.30)	10 (0.39)	9 (0.22)		
Unknown	2927 (95.21)	1292 (95.27)	1635 (95.16)		
Chronic hepatitis C, n (%)				χ^2^ = 0.069	0.966
Negative	3110 (98.29)	1369 (98.31)	1741 (98.27)		
Positive	39 (1.10)	22 (1.05)	17 (1.14)		
Unknown	31 (0.61)	17 (0.64)	14 (0.59)		
BMI, n (%)				χ^2^ = 369.964	< 0.001
Overweight/normal weight	851 (27.89)	709 (53.90)	142 (6.54)		
Overweight/obesity	2329 (72.11)	699 (46.10)	1630 (93.46)		
Carbohydrate (g), n (%)				χ^2^ = 3.290	0.193
< 188.30	1145 (33.00)	501 (35.13)	644 (31.26)		
188.30–281.09	1022 (33.99)	449 (33.65)	573 (34.28)		
> 281.09	1013 (33.00)	458 (31.22)	555 (34.46)		
Fiber (g), n (%)				χ^2^ = 0.671	0.715
< 11.15	1157 (32.86)	504 (31.97)	653 (33.58)		
11.15–18.4	975 (34.12)	432 (35.02)	543 (33.37)		
> 18.4	1048 (33.02)	472 (33.00)	576 (33.04)		
CFR, n (%)				χ^2^ = 4.967	0.083
< 12.58	1023 (33.06)	466 (36.03)	557 (30.61)		
12.58–19.91	1065 (33.80)	452 (33.38)	613 (34.14)		
> 19.91	1092 (33.15)	490 (30.59)	602 (35.25)		
Energy, n (%)				χ^2^ = 15.738	< .001
< 1709.00	1193 (33.00)	539 (35.37)	654 (31.05)		
1709.00–2481.31	1024 (33.99)	458 (36.14)	566 (32.23)		
≥ 2481.31	963 (33.01)	411 (28.49)	552 (36.71)		
Protein, n (%)				χ^2^ = 2.413	0.299
Deficient	354 (10.83)	170 (11.89)	184 (9.96)		
Sufficient	2808 (88.72)	1230 (87.63)	1578 (89.61)		
Excess	18 (0.45)	8 (0.48)	10 (0.43)		
Fat, n (%)				χ^2^ = 0.445	0.800
Deficient	138 (4.17)	68 (4.52)	70 (3.88)		
Sufficient	1258 (40.13)	576 (40.38)	682 (39.92)		
Excess	1784 (55.70)	764 (55.10)	1020 (56.19)		

*MAFLD* Metabolic dysfunction-associated fatty liver disease, *GED* General education development, *AA* Associate, *ALT* Alanine aminotransferase, *ALP* Alkaline phosphatase, *AST* Aspartate aminotransferase, *GGT* Gamma-glutamyl transferase, *BMI* Body mass index, *CFR* Carbohydrate to fiber ratio, *MET* Metabolic equivalent, *SE* Standard error

### Association between CFR and MAFLD

After adjusting for age, gender, race, education, marital status, smoking, physical activity, sedentary time, diabetes, hypertension, dyslipidemia, ALT, ALP, AST, GGT, BMI, and energy, a dietary fiber intake of 11.15–18.40 g was associated with significantly lower odds of MAFLD compared with a fiber intake < 11.15 g (OR = 0.69, 95% CI 0.52–0.91, *P* = 0.011). In contrast to a dietary CFR < 12.58, a CFR > 19.91 was associated with significantly higher odds of MAFLD (OR = 1.59, 95% CI 1.11–2.26, *P* = 0.013) (Table 2).

**Table 2:** Association between CFR and MAFLD

Variables	Model I		Model II	
	OR (95% CI)	*P*	OR (95% CI)	*P*
Carbohydrate (g), n (%)				
< 188.30	Ref		Ref	
188.30–281.09	1.14 (0.91–1.44)	0.232	1.30 (0.89–1.92)	0.166
> 281.09	1.24 (0.98–1.57)	0.069	1.32 (0.76–2.30)	0.307
Fiber (g), n (%)				
< 11.15	Ref		Ref	
11.15–18.4	0.91 (0.73–1.13)	0.379	0.69 (0.52–0.91)	0.011
> 18.4	0.95 (0.74–1.24)	0.707	0.69 (0.43–1.13)	0.134
CFR, n (%)				
< 12.58	Ref		Ref	
12.58–19.91	1.20 (0.94–1.55)	0.142	1.20 (0.87–1.66)	0.258
> 19.91	1.36 (1.01–1.84)	0.049	1.59 (1.11–2.26)	0.013

Model I, a univariate model; Model II, a multivariate model, adjusted for age, gender, race, education, marital status, smoking, physical activity, sedentary time, diabetes, hypertension, dyslipidemia, ALT, ALP, AST, GGT, BMI, and energy

*CFR* Carbohydrate to fiber ratio, *MAFLD* Metabolic dysfunction-associated fatty liver disease, *ALT* Alanine aminotransferase, *ALP* Alkaline phosphatase, *AST* Aspartate aminotransferase, *GGT* Gamma-glutamyl transferase, *BMI* Body mass index, *OR* Odds ratio, *CI* Confidence level, *Ref* Reference

### Association between CFR and MAFLD in subpopulations

#### Gender

For males, a dietary fiber intake of 11.15–18.40 g was associated with significantly lower odds of MAFLD than a fiber intake < 11.15 g (OR = 0.49, 95% CI 0.30–0.78, *P* = 0.005). Compared with females with a dietary CFR < 12.58, those with a CFR > 19.91 had significantly increased odds of MAFLD (OR = 1.90, 95% CI 1.90–2.78, *P* = 0.002) (Table 3).

**Table 3:** Association between CFR and MAFLD in subpopulations

Variables	OR (95%CI)	*P*	OR (95%CI)	*P*
Subgroup I: Gender	Male		Female	
Carbohydrate (g)				
< 188.30	Ref		Ref	
188.30–281.09	1.26 (0.74–2.15)	0.383	1.33 (0.89–1.97)	0.155
> 281.09	1.59 (0.87–2.91)	0.124	0.92 (0.42–2.01)	0.826
Fiber (g)				
< 11.15	Ref		Ref	
11.15–18.4	0.49 (0.30–0.78)	0.005	0.94 (0.63–1.39)	0.745
> 18.4	0.60 (0.33–1.07)	0.079	0.67 (0.37–1.23)	0.191
CFR				
< 12.58	Ref		Ref	
12.58–19.91	1.24 (0.68–2.27)	0.461	1.28 (0.76–2.14)	0.342
> 19.91	1.48 (0.89–2.46)	0.123	1.90 (1.30–2.78)	0.002
Subgroup II: Age	Age < 65 years		Age ≥ 65 years	
Carbohydrate (g)				
< 188.30	Ref		Ref	
188.30–281.09	1.34 (0.87–2.06)	0.170	0.94 (0.43–2.08)	0.878
> 281.09	1.48 (0.72–3.07)	0.277	0.73 (0.25–2.16)	0.559
Fiber (g)				
< 11.15	Ref		Ref	
11.15–18.4	0.70 (0.50–0.98)	0.036	0.70 (0.29–1.69)	0.407
> 18.4	0.81 (0.48–1.38)	0.429	0.44 (0.14–1.42)	0.163
CFR				
< 12.58	Ref		Ref	
12.58–19.91	1.15 (0.83–1.60)	0.377	1.32 (0.71–2.42)	0.364
> 19.91	1.54 (1.06–2.23)	0.025	1.61 (0.70–3.70)	0.246
Subgroup III: Diabetes	Diabetes = No		Diabetes = Yes	
Carbohydrate (g)				
< 188.30	Ref		Ref	
188.30–281.09	1.35 (0.90–2.03)	0.145	1.01 (0.50–2.04)	0.969
> 281.09	1.37 (0.74–2.52)	0.304	1.70 (0.37–7.79)	0.482
Fiber (g)				
< 11.15	Ref		Ref	
11.15–18.4	0.61 (0.46–0.81)	0.002	2.18 (1.13–4.20)	0.022
> 18.4	0.64 (0.38–1.07)	0.087	1.22 (0.42–3.50)	0.701
CFR				
< 12.58	Ref		Ref	
12.58–19.91	1.31 (0.95–1.80)	0.094	0.68 (0.23–2.00)	0.471
> 19.91	1.78 (1.27–2.51)	0.002	0.62 (0.19–2.10)	0.430
Subgroup IV: Hypertension	Hypertension = No		Hypertension = Yes	
Carbohydrate (g)				
< 188.30	Ref		Ref	
188.30–281.09	1.15 (0.62–2.13)	0.648	1.47 (0.85–2.53)	0.160
> 281.09	1.68 (0.64–4.42)	0.279	1.16 (0.62–2.17)	0.622
Fiber (g)				
< 11.15	Ref		Ref	
11.15–18.4	0.67 (0.38–1.20)	0.172	0.77 (0.47–1.24)	0.268
> 18.4	0.61 (0.25–1.46)	0.252	0.79 (0.48–1.29)	0.332
CFR				
< 12.58	Ref		Ref	
12.58–19.91	1.39 (0.73–2.67)	0.305	0.99 (0.63–1.54)	0.948
> 19.91	1.93 (1.02–3.68)	0.045	1.39 (0.81–2.38)	0.218

For Subgroup I, age, race, education, marital status, smoking, physical activity, sedentary time, diabetes, hypertension, dyslipidemia, ALT, ALP, AST, GGT, BMI, and energy were adjusted for; For Subgroup II, gender, race, education, marital status, smoking, physical activity, sedentary time, diabetes, hypertension, dyslipidemia, ALT, ALP, AST, GGT, BMI, and energy were adjusted for; For Subgroup III, age, gender, race, education, marital status, smoking, physical activity, sedentary time, hypertension, dyslipidemia, ALT, ALP, AST, GGT, BMI, and energy were adjusted for; For Subgroup IV, age, gender, race, education, marital status, smoking, physical activity, sedentary time, diabetes, dyslipidemia, ALT, ALP, AST, GGT, BMI, and energy were adjusted for

*CFR* Carbohydrate to fiber ratio, *MAFLD* Metabolic dysfunction-associated fatty liver disease, *ALT* Alanine aminotransferase, *ALP* Alkaline phosphatase, *AST* Aspartate aminotransferase, *GGT* Gamma-glutamyl transferase, *BMI* Body mass index, *OR* Odds ratio, *CI* Confidence level, *Ref* Reference

#### Age

Among individuals aged < 65 years, dietary fiber intake of 11.15–18.40 g was associated with lower odds of MAFLD compared to participants with fiber intake < 11.15 g (OR = 0.70, 95% CI 0.50–0.98, *P* = 0.036). And a dietary CFR > 19.91 was associated with significantly higher odds of MAFLD than a dietary CFR < 12.58 (OR = 1.54, 95% CI 1.06–2.23, *P* = 0.025) (Table 3).

#### Comorbidity

For participants without diabetes, a dietary fiber intake of 11.15–18.40 g was associated with significantly decreased odds of MAFLD compared with a fiber intake < 11.15 g (OR = 0.64, 95% CI 0.46–0.81, *P* = 0.002), and a dietary CFR > 19.91 was associated with significantly elevated odds of MAFLD than a CFR < 12.58 (OR = 1.78, 95% CI 1.27–2.51, *P* = 0.002). In participants without hypertension, a dietary CFR > 19.91 was associated with significantly increased odds of MAFLD versus a CFR < 12.58 (OR = 1.93, 95% CI 1.02–3.68, *P* = 0.045) (Table 3).

## Discussion

At present, MAFLD is a significant health issue, and it raises the risk of end-stage liver disease, hepatocellular carcinoma, mortality, and liver transplantation, and brings about extrahepatic consequences, such as cardiometabolic disease and cancers [3, 27]. Dietary CFR, consisting of two dietary modifiable factors, carbohydrates and fiber, is used to evaluate overall carbohydrate quality in the diet [28]. This study explored the association between CFR and MAFLD for the first time, and illustrated that a higher CFR was associated with significantly higher odds of MAFLD.

A higher dietary CFR, indicating a lower carbohydrate quality diet (increased intake of processed or refined foods), was reported to be associated with a greater risk of T2DM [29]. Fontanelli et al. [17] used a CFR ≤ 10:1 to identify grain foods with higher nutritional quality, and the intake of these foods was negatively correlated with atherogenic dyslipidemia and insulin resistance. CFR was associated with metabolic syndrome among patients with T2DM [16]. Hypertensive patients with a higher CFR were shown to have worse control of blood pressure [18]. The current study assessed the relationship between dietary carbohydrates, fiber and CFR and the odds of MAFLD. It was illustrated that a moderate intake of fiber was associated with lower odds of MAFLD compared with a low intake of fiber. And high CFR (> 19.91) was related to higher odds of MAFLD. Similarly, Zhu et al. [30] concluded that raising dietary fiber intake could provide greater benefits for preventing NAFLD. The association between high dietary fiber and lower odds of NAFLD was also exhibited by prior evidence [19, 31, 32].

For possible mechanisms, dietary fiber intake may delay gastric emptying and lower the levels of postprandial blood glucose [33]. The short-chain fatty acids (SCFAs) produced by the gut microbiota through dietary fiber fermentation promote energy consumption and lipid oxidation via an adenosine monophosphate-activated protein kinase (AMPK) dependent approach, which may be associated with decreased odds of NAFLD [34, 35]. SCFAs can also primarily prevent NAFLD by regulating inflammation [34]. In assessing the risk of chronic illness, there has been a greater emphasis on carbohydrate quality rather than quantity [28]. Since it integrates the relative contributions of starch and sugar with dietary fiber and is easier for the public to understand, dietary CFR has been shown to be one of the simplest and most efficient ratios used to estimate carbohydrate quality [36]. In this study, a higher CFR was demonstrated to be associated with higher odds of MAFLD, indicating that the intake of foods with higher carbohydrate quality may protect against the development of MAFLD. The beneficial effect of high dietary fiber may contribute to the association between high CFR and higher odds of MAFLD. Besides, low carbohydrates may reduce insulin resistance and further improve endothelial function and inflammation [37], thereby possibly relating to low odds of MAFLD.

Among females, or individuals aged < 65 years, without diabetes or without hypertension, high CFR was associated with higher odds of MAFLD. The prevalence of MAFLD is rising globally [38]. The difference of the association between CFR and MAFLD in men and women may be attributed to differences in sex hormone levels and sexual hormone-specific gene expression [39]. Visceral fat accumulation in specific subpopulations may be a reason for the association discrepancies. More attention could be paid to the dietary carbohydrate quality of these people, which may assist in the management of MAFLD.

The present study used a nationally representative sample from the NHANES database which adopts multi-stage sampling. As indicated, people having a higher carbohydrate quality may have lower odds of MAFLD. Individuals should have an awareness of caring about the quality of carbohydrates in order to take relevant measures to timely prevent and control MAFLD. Consuming relatively more whole grains and vegetables may be a useful measure. More prospective and randomized controlled studies are needed to determine the effect of different CFR on liver health. And identified the most appropriate CFR provided dietary recommendations for the prevention of MAFLD and metabolic diseases. Some limitations should be acknowledged. First, this study had a cross-sectional design, and thus causality could not be determined, which necessitates cohort studies to assess the causal relationship between CFR and MAFLD. Second, dietary intakes of carbohydrates and fiber were evaluated via a single 24-h dietary recall interview, which may have been affected by recall bias. And a person’s long-term diet intake was not well represented by the 24-h recall. Finally, this study used data on the American population, which may have limited generalizability.

## Conclusion

A higher CFR was associated with significantly greater odds of MAFLD. This association persisted in females, or individuals aged < 65 years, without diabetes or without hypertension. Future investigations are warranted to verify the findings.

## Data Availability

All data generated or analyzed during this study are available from The NHANES.

## Abbreviations

CFR: Carbohydrate to fiber ratio
MAFLD: Metabolic dysfunction-associated fatty liver disease
NAFLAD: Non-alcoholic fatty liver disease
NHANES: National Health and Nutrition Examination Survey
ORs: Odds ratios
CIs: Confidence levels
NCHS: National Center for Health Statistics
CAP: Controlled attenuation parameter (CAP)
T2DM: Type 2 diabetes mellitus
